# Asexuality Associated with Marked Genomic Expansion of Tandemly Repeated rRNA and Histone Genes

**DOI:** 10.1093/molbev/msab121

**Published:** 2021-04-22

**Authors:** Kyle E McElroy, Stefan Müller, Dunja K Lamatsch, Laura Bankers, Peter D Fields, Joseph R Jalinsky, Joel Sharbrough, Jeffrey L Boore, John M Logsdon, Maurine Neiman

**Affiliations:** 1 Ecology, Evolutionary, and Organismal Biology, Iowa State University, Ames, IA, USA; 2 Department of Biology, University of Iowa, Iowa City, IA, USA; 3 Institute of Human Genetics, Munich University Hospital, Ludwig-Maximilians University, Munich, Germany; 4 Research Department for Limnology, University of Innsbruck, Mondsee, Mondsee, Austria; 5 Division of Infectious Diseases, University of Colorado—Anschutz Medical Campus, Aurora, CO, USA; 6 Department of Environmental Sciences, Zoology, University of Basel, Basel, Switzerland; 7 Biology Department, New Mexico Institute of Mining and Technology, Socorro, NM, USA; 8 Department of Biology, Colorado State University, Fort Collins, CO, USA; 9 Providence St. Joseph Health and Institute for Systems Biology, Seattle, WA, USA; 10 Department of Gender, Women's, and Sexuality Studies, University of Iowa, Iowa City, IA, USA

**Keywords:** asexual reproduction, genome evolution, histone genes, repetitive sequence, ribosomal RNA genes

## Abstract

How does asexual reproduction influence genome evolution? Although is it clear that genomic structural variation is common and important in natural populations, we know very little about how one of the most fundamental of eukaryotic traits—mode of genomic inheritance—influences genome structure. We address this question with the New Zealand freshwater snail *Potamopyrgus antipodarum*, which features multiple separately derived obligately asexual lineages that coexist and compete with otherwise similar sexual lineages. We used whole-genome sequencing reads from a diverse set of sexual and asexual individuals to analyze genomic abundance of a critically important gene family, rDNA (the genes encoding rRNAs), that is notable for dynamic and variable copy number. Our genomic survey of rDNA in *P. antipodarum* revealed two striking results. First, the core histone and 5S rRNA genes occur between tandem copies of the 18S–5.8S–28S gene cluster, a unique architecture for these crucial gene families. Second, asexual *P. antipodarum* harbor dramatically more rDNA–histone copies than sexuals, which we validated through molecular and cytogenetic analysis. The repeated expansion of this genomic region in asexual *P. antipodarum* lineages following distinct transitions to asexuality represents a dramatic genome structural change associated with asexual reproduction—with potential functional consequences related to the loss of sexual reproduction.

##  

Reproductive mode is one of the most important and variable eukaryotic traits and is inherently linked to critical population genetic parameters (e.g., effective population size, heterozygosity, gene flow). Accordingly, reproductive mode is expected to play a fundamental role in driving evolutionary processes. Transitions from sexual to asexual modes of reproduction offer an especially powerful means to explore the impact of reproductive mode on evolution and can illuminate the solution to the long-standing “paradox” of why sexual reproduction is so common (Neiman et al. 2017).

Recent sequencing advances have allowed us to test major hypotheses for sex by evaluating whether asexual reproduction leaves clear genomic signatures (Glémin et al. 2019; Jaron et al. 2021). Consistent with theoretical expectations of mutation accumulation in asexuals (e.g., Muller’s ratchet [Muller 1964; Felsenstein 1974; Lynch 2007]), higher loads of likely deleterious mutations have been observed in many asexual taxa relative to their sexual counterparts (Hollister et al. 2015; Lovell et al. 2017; Bast et al. 2018; Sharbrough et al. 2018).

By contrast, we know very little about whether and how reproductive mode influences genomic architecture (e.g., gene copy number [Jaron et al. 2021]). This question is particularly pressing given the increasing evidence for the commonness and adaptive relevance of structural variation in natural populations (Wellenreuther and Bernatchez 2018; Catanach et al. 2019; Lucek et al. 2019; Wellenreuther et al. 2019; Dorant et al. 2020). Indeed, gene copy number variation was recently highlighted as an underappreciated potential source of genetic variation for asexuals (Castagnone-Sereno et al. 2019); the apparent link between asexuality and polyploidy supports that idea (Otto and Whitton 2000). In the absence of meiosis and associated homologous chromosome pairing, asexual taxa might also be particularly prone to the generation and persistence of structural changes. Although extensive gene duplication (Richards et al. 2010; Colbourne et al. 2011) and copy number variation (Hardigan et al. 2016) have been reported in asexual taxa, direct comparisons of copy number variation between closely related sexual and asexual lineages are rare (but see, e.g., Aliyu et al. 2013). The absence of this information comprises a critical gap in our understanding of how sexual versus asexual reproduction influences the evolution of genome structure.

Here, we compare copy number variation between conspecific and otherwise similar sexual and asexual individuals. Ribosomal RNA genes (rDNA) are among the most important genes for cellular life and are notably prone to genomic amplification and contraction (Gorokhova et al. 2002; Prokopowich et al. 2003; Stults et al. 2008; Nelson et al. 2019), such that rDNA copy number can vary by orders of magnitude across and within taxa (Gibbons et al. 2015). The dynamic nature of rDNA makes it an excellent setting to evaluate a potential relationship between gene copy number variation and reproductive mode because we can expect standing variation and ongoing changes in copy number from these sequences.

To test the hypothesis that changes in reproductive mode are associated with altered genomic architecture, we estimated rDNA copy number in sexually versus asexually transmitted genomes using data from the in-progress reference genome project for *Potamopyrgus antipodarum*, along with whole-genome sequencing of 16 obligately asexual and 10 obligately sexual *P. antipodarum* lineages collected from natural populations (supplementary table S1, Supplementary Material online, fig. 1). This New Zealand freshwater snail is a textbook model system for the study of sex because natural populations of *P. antipodarum* are characterized by numerous distinct transitions to obligate asexuality from the ancestral obligate sexual state (Dybdahl and Lively 1995; Paczesniak et al. 2013) and the frequent coexistence of ecologically and phenotypically similar sexual and asexual individuals (Lively 1987; Jokela et al. 1997). Nuclear genotyping studies of offspring produced by female *P. antipodarum* collected from a variety of all- or nearly all-female populations in Europe and New Zealand (Phillips and Lambert 1989; Hauser et al. 1992) or clonal lineages cultured in the laboratory (Million et al. 2021) have invariably indicated reliable preservation of multilocus nuclear genotypes, suggesting strongly that asexual female *P. antipodarum* are obligately apomictic.

**Fig. 1:**
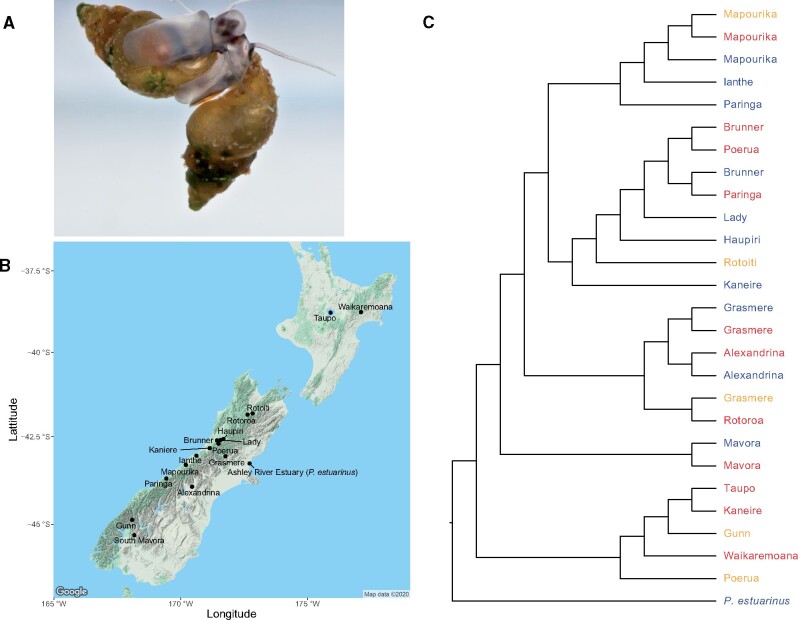
*Potamopyrgus antipodarum* as a model for maintenance of sex and consequences of asexuality. (*A*) Two *P. antipodarum* underwater. (*B*) Collection sites (lakes) in New Zealand for the lineages included in this study of *P. antipodarum* and the estuary for *Potamopyrgus estuarinus*. Map with locations was produced in R with ggmap (Kahle and Wickham 2013) (*C*) Maximum-likelihood cladogram of *P. antipodarum* lineages and *P. estuarinus* used in this study, produced with RAxML (Stamatkis 2014) based on 70,437 nuclear SNPs (Bankers 2017; Jalinsky et al. 2020). Lake names refer to sequenced *P. antipodarum*. Tips are colored by reproductive mode and ploidy: blue (sexual and diploid), red (asexual and triploid), yellow (asexual and tetraploid).

This unusual situation enables us to treat distinct asexual lineages as powerful separate natural experiments into the consequences of the absence of sex. Because sexual *P. antipodarum* are diploid whereas asexuals are either triploid or tetraploid (Neiman et al. 2011; ploidy elevation almost certainly represents autopolyploidy, Dybdahl and Lively 1995; Paczesniak et al. 2013), we can also use this ploidy variation among asexuals to both perform an assessment of the effects of ploidy elevation on rDNA copy number and in part decouple effects of ploidy from reproductive mode.

## Results and Discussion

### Unique Genomic Architecture for rDNA and Histones in *Potamopyrgus*

**Fig. 2:**
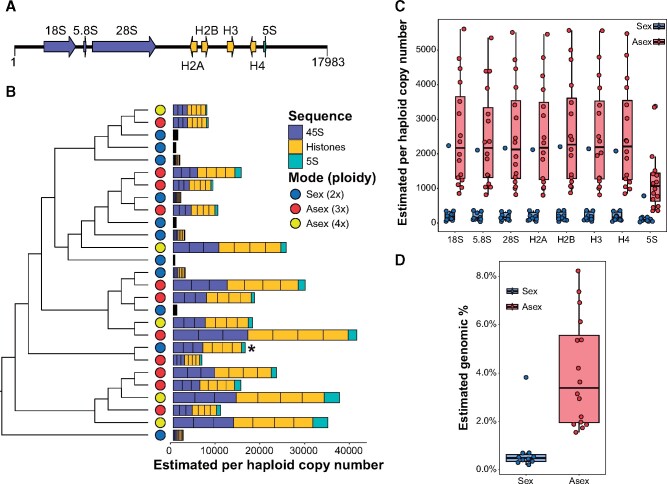
(*A*) Organization of rRNA genes (purple for 45S, the precursor rRNA of the final products of 18S, 5.8S, and 28S rRNAs, and teal for 5S) and core histones (orange) in *Potamopyrgus*. Arrows indicate the relative transcriptional orientations, and the scale represents genomic coordinates for one arbitrary copy of the rDNA–histone locus. Locus schematic produced with Illustrator for Biological Sciences (Liu et al. 2015). (*B*) Estimated per-haploid copy number, based on sequence depth normalized by single-copy regions, for each lineage of *Potamopyrgus antipodarum*, and the obligately sexual outgroup *Potamopyrgus estuarinus* (bottommost branch). The order of genes in the schematic in (*A*) reflects the order of copy-number values in the stacked-bar plot. The lineages in (*B*) are arranged as in figure 1 (* possible asexual diploid from South Mavora lake). Asexual lineages (red) of *P. antipodarum* have markedly higher abundance of rDNA–histone sequence in their genomes than sexuals (blue) (MWU *P *<* *0.0001; phylogenetic GLM *P *<* *0.01). Tukey’s boxplots with all data points shown of (*C*) estimated copy-number for each gene in the rDNA–histone array for sexual (*n* = 10 lineages) and asexual lineages (*n* = 16 lineages) and (*D*) estimated genomic proportion of rDNA–histone repeats in sexual versus asexual lineages, based on the % of sequence reads that map to one rDNA–histone locus divided by the number of reads mapping to the entire genome assembly. Asexuals have higher genomic proportions of rDNA–histone sequence than sexuals (MWU *P *<* *0.0001).

We identified rRNA gene sequences in the *P. antipodarum* genome assembly with RNAmmer (Lagesen et al. 2007), which uses hidden Markov models trained from the European ribosomal RNA database project to identify rRNA elements (Quast et al. 2013). The rRNA genes form two RNA subunits in a eukaryotic ribosome, the large (LSU) and small (SSU) subunits. The 5.8S, 28S, and 5S genes contribute to the LSU, whereas the 18S gene contributes the RNA component to the SSU. Eukaryotes usually have multiple copies of 18S–5.8S–28S (referred to as “45S” in metazoans) units in tandem clusters, along with multiple separate 5S clusters. Our survey of rDNA in the *P. antipodarum* genome revealed that copies of the four core histones (H2A, H2B, H3 and H4) and the 5S rRNA gene are located between copies of the canonical 45S rRNA locus (supplementary table S2, Supplementary Material online, fig. 2*A*). Together, this rDNA–histone sequence is tandemly repeated in the *P. antipodarum* genome. The striking similarity between estimated histone and rDNA copy number that we observe in *Potamopyrgus estuarinus* and *P. antipodarum* (fig. 2*B*, supplementary fig. S2, Supplementary Material online) indicates that this multigene family architecture is ancestral to the *Potamopyrgus* genus.

In many animal lineages, histones are organized into tandem quartets containing only the four core histones or into quintets that also include the linker H1 gene. Mollusks feature a wide range of histone arrangements and associations between different repeat families. For example, the mussel *Mytilus galloprovincialis* has three different histone organizations, one of which is the histone quintets linked with 5S repeats (Eirin Lopez et al. 2009). Associations of 5S with either 45S or histones have evolved in a variety of taxa (Drouin and Moniz de Sá 1995), including in at least one other mollusk, a slug with colocalizing 45S and 5S genes in its genome (Vitturi et al. 2004). The union of these three distinct repetitive gene families in *Potamopyrgus* appears to be uniquely derived. Two congeneric weevil species exhibit remarkably convergent architectures to *P. antipodarum*, in which histone quintets are located between 45S copies (Roehrdanz et al. 2010), which raises the intriguing question of whether this architectural conformation is adaptive in some way. *Potamopyrgus antipodarum* differs from this organization in these weevils only by its inclusion of 5S and absence of H1 in the repeated sequence.

### Genomic Copy Number Estimates of rDNA–Histone Repeats

We used Illumina paired-end whole-genome sequence data from 16 asexual (11 triploid and 5 tetraploid) and 10 sexual lineages along with one female *P. estuarinus* (fig. 1; see [Bankers 2017; Jalinsky et al. 2020] for description of sampling, sequencing, and phylogenetic reconstruction) to estimate per-haploid copy number based on read depth for each component of the rDNA–histone locus normalized by the read depth of a set of single-copy exons (supplementary fig. S1, Supplementary Material online). There is a strong positive correlation for copy-number estimates for each of the genes in this locus (supplementary fig. S2, Supplementary Material online). In particular, our copy-number estimates for the 45S genes and histones were nearly identical within each individual. The somewhat lower copy-number estimates for 5S suggests that the *P. antipodarum* genome contains rDNA–histone loci lacking 5S. Overall, the consistency in copy-number estimates across each rDNA–histone component per sequenced lineage indicates that this tandemly repeated sequence changes copy number as an entire unit and the 45S does not independently expand or contract (fig. 2).

### Asexuals Accumulate Higher Amounts of rDNA–Histone Sequence Relative to Sexuals

Asexual lineages of *P. antipodarum* have markedly more copies of the rDNA–histone locus than sexuals (fig. 2*B* and *C*; MWU *P *<* *0.0001). We used a phylogenetic generalized linear model to evaluate whether the phylogenetic distribution of asexuality in the lineages we sampled could account for rDNA–histone accumulation rather than asexuality per se. Because asexuality has evolved on multiple separate occasions in *P. antipodarum* (Dybdahl and Lively 1995; Paczesniak et al. 2013), the strong association between rDNA–histone expansions and asexuality that this analysis revealed (*P *<* *0.01, fig. 2*B*) suggests instead that asexual reproduction is a direct or indirect source of these expansions. Our copy number estimates for the obligately sexual *P. estuarinus* fall within the range of sexual *P. antipodarum*, providing another line of evidence that the higher rDNA–histone abundance in asexual versus sexual lineages is indicative of expansion of this sequence in asexuals, rather than contraction in sexual lineages.

An apparent exception to the overall pattern of higher rDNA–histone copy number in asexuals provides even more support for our conclusion: a female snail with a diploid nuclear genome size (and, thus, assigned “sexual” status) collected from South Mavora lake represents the only sexual sample with rDNA–histone copy number in the range of the asexual snails (the outlier for sexual lineages within the asexual range in fig. 2*C* and *D*). The occasional observation of *P. antipodarum* with diploid genome size from lakes like South Mavora (New Zealand, South Island) where the *P. antipodarum* are virtually all female (and, thus, almost certainly all asexual) might reflect asexual lineages that have not experienced ploidy elevation (Neiman et al. 2011). We speculate that this South Mavora snail could very well represent one of these diploid asexuals and, thus, bears the relatively high rDNA–histone copy number that characterizes asexual *P. antipodarum*.

We did not observe a difference in rDNA–histone genomic abundance between triploid and tetraploid asexuals (supplementary fig. S3, Supplementary Material online, adjusted *P *>* *0.5 in triploid vs. tetraploid comparisons). This result suggests that asexuality per se is the driving factor for this sequence expansion instead of ploidy elevation, though we cannot yet formally exclude a potential role for the latter. This conclusion regarding asexuality finds additional support from our observations that asexual *P. antipodarum* rDNA–histone copy number can be 10× higher than closely related and sympatric sexuals (e.g., snails from Lake Grasmere, fig. 2*B*) and that closely related sympatric triploid and tetraploid asexual lineages (e.g., snails from Lake Mapourika, fig. 2*B*) have nearly identical copy number. Taken together, these data indicate that effects of ploidy elevation on rDNA–histone expansion, if any, will likely be more subtle than asexuality.

### Consistent rDNA–Histone Copy Numbers within Natural Clonal Lineages

Asexual *P. antipodarum* collected blind to genotype from natural populations but subsequently found to be members of the same clonal lineage have nearly identical rDNA–histone copy number that are easily distinguishable from other clones (supplementary fig. S4, Supplementary Material online). We collected additional *P. antipodarum* from two New Zealand lakes (Grasmere, Haupiri), characterized ploidy with flow cytometry, and then genotyped 92 triploid asexuals from Grasmere and 83 triploids from Haupiri with a 46-SNP array previously used in Verhaegen et al. (2018). We then assigned clonal genotypes to each individual using GenoDive (Meirmans and Van Tienderen 2004), identifying 45 different clonal genotypes from Grasmere and 19 from Haupiri. These two lakes did not share any clones, reflecting the established population structure of *P. antipodarum* and locally derived asexual lineages (Paczesniak et al. 2013). Finally, we selected three snails from each of four clonal genotypes, two genotypes from each lake, for Illumina whole-genome sequencing. We used these data to estimate copy number per each of the histone and rDNA genes, which revealed strikingly similar copy numbers within clonal lineages and markedly different copy numbers between distinct clones (supplementary fig. S4, Supplementary Material online). These genotype-informed copy number results indicate that rDNA–histone copy number is reliably heritable on the very short evolutionary timespans likely to characterize individuals that share genotypes across dozens of highly variable SNP loci. For each of the two lakes, one genotype had nearly twice the rDNA–histone copy number of the other. These marked across-clone differences in rDNA–histone abundance demonstrate that this genomic trait can vary widely within asexual assemblages of *P. antipodarum*, which is in turn consistent with a relatively high rate of change for rDNA–histone tandem repeat copy number as asexual lineages diverge. These rDNA–histone abundance observations from asexual *P. antipodarum* are broadly reminiscent of rDNA copy number dynamics observed in *Arabidopsis thaliana* mutation accumulation lines, where copy number diverged across lines within 30 generations despite reliable heritability from one generation to the next (Rabanal et al. 2017). Indeed, a rapid pace of rDNA copy number change in asexual *P. antipodarum* would drive the wide variation across rDNA copy number in asexual *P. antipodarum* and explain why clonal genotype appears to be a better predictor of rDNA–histone copy number than lake population.

### Expansion of rDNA–Histone Sequence in Asexuals May Influence Genome Size Variation

**Fig. 3:**
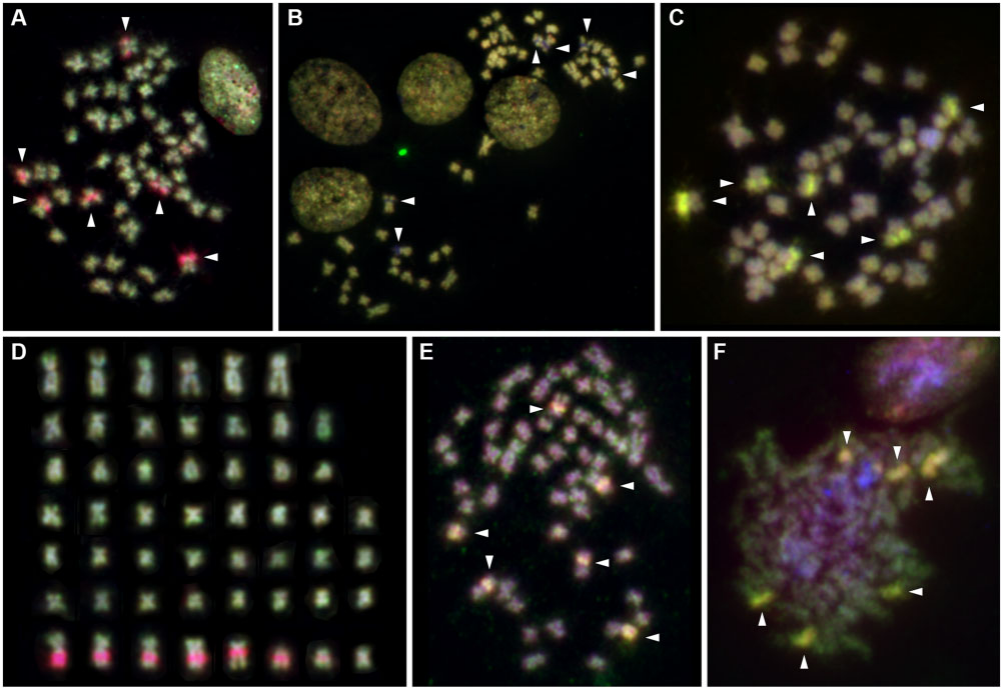
Cytogenetic analysis in *Potamopyrgus antipodarum*. (*A*–*D*) representative FISH images from comparative genomic hybridization (CGH) experiments. (*A*) Comparison of diploid sexual female MF1 (green) versus triploid asexual female (red) demonstrates marked copy-number overrepresentation of six major clusters of rDNA/histone gene cluster-related copy number variation in asexual genomes as indicated by red FISH staining in these regions (arrowheads). (*B*) and (*C*) comparison of individuals with same ploidy levels showed much more balanced genomes. (*B*) Two diploid sexual males, individual M1 in green versus B1 in red, each with extremely low copy numbers in rDNA copy-number variation regions, as indicated by hybridization gaps (arrowheads). (*C*) Triploid asexual female B3 in red versus triploid asexual male B3 in green, both with equally very high copy number as indicated by equally high relative hybridization intensities and a yellow FISH staining (arrowheads). (*D*) Karyotype analysis based on CGH pattern and chromosome morphology revealed 52 chromosomes in the majority of cells and six major and two minor rDNA/histone gene cluster size-related copy-number variation (red, bottom panel). (*E*) and (*F*) Colocalization analysis by sequential CGH and FISH with 28S rDNA and histone gene probes mapped (*E*) a *Drosophila melanogaster* BAC clone comprising clusters of histone genes (green) and (*F*) a *P. antipodarum* 28S rDNA PCR product (green) to the above described regions of clustered copy-number variation (red). Regions of colocalization between rDNA/histone gene loci and copy-number variation detected by CGH showed a yellow blended color (arrowheads).

We here asked whether the marked rDNA–histone copy number expansion that we observed in asexual *P. antipodarum* drives variation in the proportional contribution of rDNA–histone sequence to the genome. We mapped the genomic reads of each lineage to one copy of the approximately 13 kb rDNA–histone locus and divided the number of mapped reads by the number of reads that map to the entire genome assembly. We used this value to represent the proportion of the genome comprised of rDNA–histone sequence. As expected, the asexuals had distinctly higher genomic proportions of rDNA–histone DNA (*P *<* *0.0001, fig. 3*B*). All told, expansion of rDNA–histone sequences in asexuals represents a dramatic change in genome composition: rDNA–histone sequence comprised 0.21–0.71% of sexual genomes (disregarding the South Mavora diploid, 3.82%) and 1.68–8.22% of asexual genomes (fig. 3*B*). These data are consistent with multiple previous lines of evidence pointing to wide intra-ploidy genome size variation in *P. antipodarum* (Wallace 1992; Neiman et al. 2011; Paczesniak et al. 2013; Million et al. 2021). Indeed, genome-size variation across certain accessions of *A. thaliana* is largely due to differences in rDNA abundance (Long et al. 2013). Accordingly, we suggest that the wide range in rDNA–histone genomic content we observed among asexual *P. antipodarum* may play a key role in observed intraploidy genome size variation in this species (Neiman et al. 2011; Million et al. 2021).

### Cytogenetic Methods Confirm rDNA–Histone Increase in Asexuals

Comparative genomic hybridization (CGH), coupled with fluorescence in situ hybridization (FISH), provided an independent line of support for our bioinformatic finding of higher rDNA–histone copy number in asexual relative to sexual *P. antipodarum* (fig. 3, supplementary figs. S5 and S6, Supplementary Material online). We found that triploid asexual *P. antipodarum* genomes harbor six major clusters of 28S rDNA and histone genes. These clusters, at the resolution of FISH, are spatially colocalized and correspond to major regions of copy-number variation. We consistently detected these six chromosomes (five large submetacentric chromosomes and one medium sized acrocentric chromosome) harboring major rDNA clusters and two chromosomes (one medium sized submetacentric chromosome and one small metacentric chromosome) with minor rDNA–histone gene clusters, irrespective of the total chromosome count of 52 or 53 chromosomes. Because asexual *P. antipodarum* occasionally produce males (Neiman et al. 2012; Jalinsky et al. 2020), we included asexually produced males in full-factorial CGH analysis for copy-number variation across reproductive mode (sex vs. asex) and sex (male vs. female). We did not observe noticeable copy number differences for rDNA–histone loci across sexes or ploidy levels (supplementary fig. S6*d*, Supplementary Material online), nor did we detect entire chromosomes with an overabundance of DNA sequence only found in asexuals. Our cytogenetic results provide a separate line of evidence that the copy number differences between reproductive modes are much more pronounced than ploidy level differences in asexuals. The rDNA–histone expansions in asexual lineages are thus not likely attributable to male versus female sex, inter-individual copy number variation, extrachromosomal DNA, B chromosomes, or aneuploidies exclusive to asexual *P. antipodarum*.

### What Are the Causes and Consequences of rDNA–Histone Expansion in Asexuals?

Our discovery that asexual *P. antipodarum* repeatedly experience dramatic rDNA–histone expansions raises broadly important questions: how and why does copy number increase so markedly in asexual lineages, is this mechanism likely to be generalizable across asexual taxa, and what are the evolutionary consequences of this genomic change? Unequal crossover during meiotic homologous recombination is a common mechanism for copy number change (Hastings et al. 2009) and sequence evolution in rDNA (Eickbush and Eickbush 2007). Although we cannot formally exclude this mechanism, the likelihood that asexual *P. antipodarum* produce offspring *via* apomictic parthenogenesis (Phillips and Lambert 1989; Hauser et al. 1992; Dybdahl and Lively 1995; Million et al. 2021) suggests that a mechanism other than homologous recombination might be involved. One possibility is that rDNA copy number increase in asexual *P. antipodarum* could arise from unequal sister chromatid exchange (USCE) during apomictic recombination. Extensive change in rDNA repeat units driven by apomictic recombination has been reported in multiple asexual taxa in experimental evolution experiments (Gorokhova et al. 2002; Shufran et al. 2003). Accordingly, USCE is a plausible process for rDNA increase in asexual *P. antipodarum*, though the present data do not allow us to directly support this model or rule out other mechanisms. Experiments targeted at characterizing the mechanisms driving rDNA expansion in *P. antipodarum* present a promising line of future research.

Natural selection also likely plays an important role in rDNA copy number evolution (Press et al. 2019). A good example of a role for selection comes from Bik et al. (2013), who measured rDNA copy number across 400 generations in *Caenorhabditis elegans* mutation accumulation lines initiated with the same rDNA copy number. This study revealed substantial accumulation of variation, averaging 2× copy number increase across the study, indicating that selection had been previously restricting rDNA accumulation (Bik et al. 2013). The distinct increase in rDNA–histone copy number in asexual *P. antipodarum* is consistent with scenarios where selective constraints on the accumulation of these sequences is reduced in asexuals or where the efficacy of selection is reduced in an asexual context (e.g., via the Hill–Robertson effect [Hill and Robertson 1966]), allowing slightly deleterious genomic elements to accumulate. The absence of meiotic chromosome pairing could also allow major copy number changes to persist in asexuals (Jaron et al. 2021). Our findings set the stage for future studies disentangling the relative contribution of direct or indirect consequences of asexual reproduction versus relaxed or less effective selection in asexual populations to the phenomenon of asexual rDNA–histone accumulation in *P. antipodarum*.

The potential consequences of rDNA–histone expansion in asexuals are wide ranging. Asexual populations are expected to accumulate higher loads of mildly deleterious mutations relative to sexuals. Whether the rDNA–histone accumulation we observed in asexual *P. antipodarum* represents a higher deleterious mutation load is unclear, though the mutational hazard hypothesis (Lynch 2007) posits that excess DNA should increase the likelihood of harmful mutations and therefore carry negative fitness consequences. The higher rDNA–histone copy numbers in asexuals could also have direct phenotypic effects. Ecological and life history traits in bacteria have clear connections to rDNA copy number (Klappenbach et al. 2000; Stevenson and Schmidt 2004; Roller et al. 2016), but relationships between rDNA copy number and specific phenotypes in eukaryotes remain largely hypothetical and unexplored (Press et al. 2019). Still, rDNA copy number variation has been associated with global gene expression in plants and animals (Paredes et al. 2011; Gibbons et al. 2014; Li et al. 2018), and we do know that the loss of rDNA copies below certain thresholds can stunt development or even be lethal (Ritossa et al. 1966; Delany et al. 1994). There is also some evidence for a positive relationship between rDNA copy number and organismal growth (Su and Delany 1998; White and McLaren 2000).

The framework for a connection between rDNA and organismal growth is described by the growth-rate hypothesis, which is based on the observation that organismal ratios of carbon, nitrogen, and phosphorus (P) are influenced heavily by the demand for P-rich rRNA to support the ribosome demand of growth (Elser et al. 2000; Weider et al. 2005). This hypothesis predicts that rapidly growing consumer organisms that are growth limited by dietary P-availability might be particularly affected by changes in rDNA abundance. Growth rate in *P. antipodarum* is sharply reduced under dietary phosphorus limitation (Tibbets et al. 2010; Neiman et al. 2013; Krist et al. 2014; Neiman and Krist 2016), and asexual *P. antipodarum* demonstrate extensive genetic and population-level variation in response to P limitation (Krist et al. 2014; Krist et al. 2017). The naturally occurring wide range of rDNA abundance we observe here coupled with the strong link of P availability to *P. antipodarum* growth presents a compelling setting to directly test predictions from the growth-rate hypothesis.

These ideas linking growth rate and rDNA are especially promising in light of the fact that two common-garden studies showed that asexual *P. antipodarum* exhibit higher growth rates, regenerate tissue more rapidly, and reach reproductive maturity at a younger age than sexuals (Krois et al. 2013; Larkin et al. 2016). Neither of these studies detected significant differences between triploid and tetraploid asexuals. Although we cannot formally exclude the possibility that there might exist relatively subtle and heretofore undetected differences between triploids and tetraploids (vs. diploids and polyploids), these results are consistent with a scenario where ploidy elevation does not fully account for the differences in life history trait expression and tissue regeneration in sexual versus asexual *P. antipodarum*. The distinctly higher copy number of rDNA–histones in asexual versus sexual *P.* a*ntipodarum* could be a contributing factor to why asexual *P. antipodarum* grow at a higher rate than their sexual counterparts, with important downstream implications for the outcome of competition between sexuals and asexuals in natural populations.

## Conclusion

Our results indicate that rDNA–histone gene accumulation is a defining genomic feature of asexual *P. antipodarum*. The wide range in rDNA–histone copy number among asexual *P. antipodarum* reveals a potentially important source of rapidly generated genetic variation that could plausibly influence ecologically relevant phenotypes. The mechanisms driving this expansion and the biological and evolutionary consequences of this dramatic genomic change present a new avenue to explore regarding the genomic consequences of asexuality in this powerful natural system. Whether rDNA expansion is a common feature in asexual organisms or simply a peculiarity of *P. antipodarum* represents a distinctly new open question concerning the effects of asexual reproduction on genome evolution.

## Materials and Methods

### *Potamopyrgus* Collection and Sequencing

For our primary investigation into genomic comparisons between sexual and asexual *P. antipodarum* lineages, we collected snails from 16 lakes and the Ashley River Estuary (for *P. estuarinus*) in New Zealand (fig. 1, supplementary table S1, Supplementary Material online). We collected additional snails from Lakes Haupiri and Grasmere for a within-population and clonal genotype-informed examination of copy number. We assessed ploidy via flow cytometry following Larkin et al. (2016) and extracted genomic DNA from female snails following a modified CHOAS phenol-chloroform extraction (Sharbrough et al. 2018; detailed protocol: “CHAOS DNA extraction with PLG tubes.docx” available at https://github.com/jsharbrough/protocols, last accessed May 5, 2021). To compare sexual and asexual genomes, we generated Illumina paired-end genome sequence data from 16 obligately asexual and 10 obligately sexual *P. antipodarum* lineages collected from natural populations, along with *P. estuarinus* (see supplementary table S1, Supplementary Material online for library preparation, sequencing platform, and read size information). For genomic comparisons within and between genotypes of triploid asexual snails, we first genotyped 92 snails from Grasmere and 83 snails from Haupiri with a 46-SNP array previously used in Verhaegen et al. (2018). We used GenoDive (Meirmans and Van Tienderen 2004) to assign clonal genotypes to each individual snail. We then generated Illumina paired-end whole genome sequence data from three individuals from each of four genoyptes, two genotypes from each lake, for a total of 12 snails (supplementary table S2, Supplementary Material online). We used FastQC v.0.11.7 (Andrews 2010) to assess read quality information before and after trimming reads with Sickle version 1.33 (Joshi and Fass 2011) for all 39 sequenced libraries.

### Identification of rDNA in *P. antipodarum* Genome Assembly

We used RNAmmer (Lagesen et al. 2007), with hidden Markov models trained from the European ribosomal RNA database project (Quast et al. 2013), to identify rRNA genes in the *P. antipodarum* genome assembly. Because the RNAmmer genomic coordinates for 28S rRNA represented the 5.8S–ITS2–28S sequence, we manually curated an arbitrary full-length representative of 28S and 5.8S separately. We used BlastX to query one arbitrary full-length representative of the associated 45S and 5S rRNA genes against the nr database on NCBI (Coordinators 2018) to search for potential coding genes included in the joint 45S–5S rRNA gene cluster. This search returned coding sequences for the core histone genes: H2A, H2B, H3, and H4. Next, we selected one arbitrary full-length sequence of the each rRNA and histone gene sequences and used BlastN (BLAST+ version 2.6.0; Camacho et al. 2009) with an *e*-value cutoff of 1*e*-50 to search these sequences against the *P. antipodarum* genome assembly and generated a gene-by-gene coordinate set of the rDNA–histone sequences (supplementary table S2, Supplementary Material online). We determined the organization of this multi-gene array and the orientation of each gene based on these BlastN results (fig. 2*A*).

### Copy Number Estimation of rDNA and Histones

We used the Benchmarking Universal Single-Copy Ortholog program (BUSCO, version 3.0.2; Waterhouse et al. 2018) with the metazoan (obd9) lineage data to identify single-copy sequences in the *P. antipodarum* genome that we would use for estimating rDNA and histone gene copy number. Our BUSCO results included 879 complete single-copy genes. Next, we collected the longest exon from each complete single-copy BUSCO gene at least 300 bp long from the Augustus (version 3.3.2; Stanke et al. 2008) gene models (626 exons met this criterion). Then we mapped reads from all 26 *P. antipodarum* lineages and *P. estuarinus* with BWA mem (Li and Durbin 2009) to these 626 sequences and quality filtered (*q* ≥ 20) the mapping results with samtools (version 1.2; Li et al. 2009). We measured the per-base coverage of these 626 exon sequences for each of the 27 lineages with bedtools (version 2.25.0; Quinlan and Hall 2010).

To generate a reliable set of sequences to use for rDNA–histone copy number estimation, we filtered down the initial set of 626 putatively single-copy exons to a subset with even sequencing coverage and single-copy status in our *Potamopyrgus* whole-genome sequence data. We calculated mean, median, and mode sequence coverage for each exon for all 27 lineages. Then we retained exons with a mean, median, and mode coverage all within 10% of each other from all 27 lineages. We found 12 exons meeting our criteria in at least 20/27 of the lineages. Two of these exons had sequence coverages well outside of the range of the ten exons (∼11× and ∼15× higher), indicating that they were high copy-number sequences and we therefore excluded them as single-copy sequences for subsequent rDNA–histone copy number estimation.

We used the final set of ten exons (7,281 bp total) with relatively even sequence coverage for all samples (supplementary fig. S1, Supplementary Material online) as our single-copy standard for estimating copy number in rDNA–histone genes. To estimate per-haploid copy number of rDNA and histone genes, we mapped reads with BWA mem (version 1.2) to each rDNA and histone gene along with the ten single-copy exons and then quality filtered (*q* ≥ 20) the mapping results with samtools (version 1.2). We measured the per-base coverage of the rDNA–histone genes and single-copy exons with bedtools (version 2.25.0). We then calculated median coverage for each rRNA and histone gene and then divided this value by the median coverage for the combined ten single-copy exons, resulting in our per-haploid genome copy-number estimation. We used the same single-copy exons to estimate rDNA–histone copy number in the additional 12 triploid individuals resequenced from Grasmere and Haupiri.

### Comparison of rDNA and Histone Copy Numbers

We used the R package PerformanceAnalytics to perform Pearson’s correlation tests between copy-number values for all rDNA and histone genes. We used a phylogenetic generalized linear model using Poisson regression with generalized estimating equations (Paradis et al. 2017) with the R package phylolm (Tung Ho and Ané 2014) to test for relationships between rDNA–histone copy number and reproductive mode (sexual vs. asexual), with the phylogenetic relationship of the lineages taken into account. Our estimated per-haploid copy number for 18S, 5.8S, 28S, H2A, H2B, H3, and H4 were all highly and positively correlated (Pearson’s *r *=* *1.00 for all pairwise comparisons). The 5S copy number showed a similar positive relationship to the other genes, but the correlation was somewhat lower in magnitude (Pearson’s *r *=* *0.88–0.90, *P *<* *0.01 in all pairwise comparisons). The rDNA–histone genes are not independent of one another, so we used Mann–Whitney *U* (MWU) tests and Kruskal–Wallis (KW) tests to compare copy number of 5.8S (as a representative of the 45S and histone gene copy number) in sexual versus asexual lineages and across diploid, triploid, and tetraploid lineages, respectively. We performed a separate comparison of 5S copy number between sexual versus asexual lineages and across ploidy levels. We applied the Benjamini–Hochberg method for multiple-test comparison in KW tests.

### Estimation and Comparison of rDNA–Histone Locus Genomic Proportion

To estimate the total and fractional contribution of rDNA–histone sequence to each genome, we extracted an arbitrary full-length copy of the entire rDNA–histone locus from the *P. antipodarum* genome assembly with samtools. Next, we used RepeatMasker (version 4.0.7; Smit et al. 2015) to remove simple repeats from this locus. We then mapped reads, with bwa mem, from every *P. antipodarum* lineage and from *P. estuarinus* to the masked rDNA–histone sequence and to the entire *P. antipodarum* genome assembly. We used samtools flagstat to count the number of reads mapping to the genome and to the single masked rDNA–histone locus. Next, we divided the number of reads mapping to the rDNA–histone locus by the total number of reads mapping to the genome to estimate genomic proportion of rDNA–histone sequence. Finally, we compared the estimated genomic proportion of rDNA–histone sequence between sexual and asexual *P. antipodarum* lineages with a MWU test.

### Cytogenetic Analysis

#### Chromosome Preparations

We prepared chromosomes from three individuals of female asexual *P. antipodarum* collected at Mondsee, Austria. The snails were kept in 0.03% colchicine solution (w/v) for 4.5 h, followed by hypotonic treatment in ddH_2_O for 40 min. The bodies from the snails were extracted from shells, fixed in methanol/acetic acid 3:1 v/v, and stored at –20 °C. The bodies were then transferred to petri dishes containing 40% (v/v) acetic acid at room temperature and sheared into the smallest possible pieces for 5 min using a scalpel. We dropped the resulting cell suspension onto a cleaned ice-cold and wet microscopic slide. A few drops of methanol/acetic acid 3:1 v/v were added immediately, and the slide was air dried and then dehydrated using the ascending ethanol series 70%/90%/100% v/v at room temperature for 3 min each.

#### DNA Probes for FISH

We established a 28S rDNA FISH probe by PCR amplification and labeling in the presence of Cy3-dUTP from a gDNA mixture of ten *P. antipodarum* individuals using forward 5′-CTC TCG TAC CGA GCA GAA TTA C-3′ and reverse 5′-GAG GAT GGA AAC CTC GCA TAG-3′ primers under the following cycling conditions: 94 °C 2 min 1× (94 °C 0.5 min/51 °C 0.5 min/74 °C 1 min) 25×, 74 °C 5 min 1×. *Drosophila melanogaster* bacterial artificial chromosome (BAC) R21L19 (chr2L: 21,421,037–21,563,821; insert 142,785 bp; Apr. 2006 assembly BDGP R5/dm3) containing histone cluster DNA sequences was used as the histone cluster FISH probe. We performed BAC DNA preparation, amplification and labeling for FISH as described (Müller et al. 2007).

We used genomic DNA for CGH experiments from 13 *P. antipodarum* individuals: four diploid sexual males and each of three sexual diploid females, triploid asexual males, and triploid asexual females. The triploid snails were all from a triploid asexual lineage isolated from Lake Heron in New Zealand in 2009. The sexual snails were from a lineage isolated from New Zealand's Lake Selfe in 2010. Genomic DNA was amplified, labeled with Biotin-dUTP, Digoxigenin-dUTP, Alexa488-dUTP, and Cy3-dUTP or TexasRed-dUTP, and prepared for CGH as previously described (Neusser et al. 2005). We combined differentially labeled genomic DNA for FISH in sets of 2–4 probes.

For each FISH experiment, we combined all labeled probes, ethanol coprecipitated with 20 µg salmon sperm DNA, and then resuspended in hybridization buffer at a final concentration of 200 ng/µl hybridization mix per probe.

#### Sequential Fluorescence In Situ Hybridization (Re-FISH) and Immuno-FISH

In order to correlate information on genomic imbalances from CGH analyses with physical mapping data of histone and 28S gene clusters, we performed sequential FISH experiments to the same microscopic slide, each round followed by repositioning and recapturing of hybridized metaphases essentially as previously described (Müller et al. 2002). In brief, for the first round of sequential FISH experiments, we denatured DNA probes at 75 °C for 5 min, added the probes to the slide with the metaphase preparation, covered the slide with a cover slip, and sealed the cover slip with rubber cement. The slide was then denatured at 75 °C for 2 min in a Hybrite (VYSIS, US) hybridization station and hybridized at 37 °C overnight. Posthybridization washes included 4 min incubation in 0.1×SSC buffer at 60 °C. Biotinylated probes were detected with Avidin-Alexa488 (Molecular Probes), and digoxigenin-labeled probes were detected with Mouse-anti-Digoxigenin-Cy5 antibody (Dianova, Germany). We performed subsequent FISH rounds as described in (Müller et al. 2002). FISH round 2 was combined with immuno-fluorescence staining using an anti-5-Methylcytosine antibody (Diagenode), to visualize hypermethylated chromosome segments. After each FISH round, we mounted the slides in Vectashield embedding medium containing DAPI (Vector Laboratories, UK) and then performed microscopic evaluation using an Axioplan 2 Imaging microscope (Zeiss, Germany) equipped with fluorescence filter sets for DAPI, DEAC, FITC, Cy3, TexasRed and Cy5. We analyzed at least ten metaphases per experiment.

## Supplementary Material

Supplementary data are available at *Molecular Biology and Evolution* online.

## Supplementary Material

msab121_Supplementary_Data
