# Supplementary material for: Asexuality Associated with Marked Genomic Expansion of Tandemly Repeated rRNA and Histone Genes
Source: Mol Biol Evol. 2021 Apr 22;38(9):3581–92. doi: 10.1093/molbev/msab121 (PMC8382920; doi:10.1093/molbev/msab121)
Supplement: msab121_Supplementary_Data [file msab121_supplementary_data.zip › McElroy_et_al_Supp_MBE_fina_4-19-21.docx]

SUPPLEMENTAL MATERIALS

Figures S1-S6


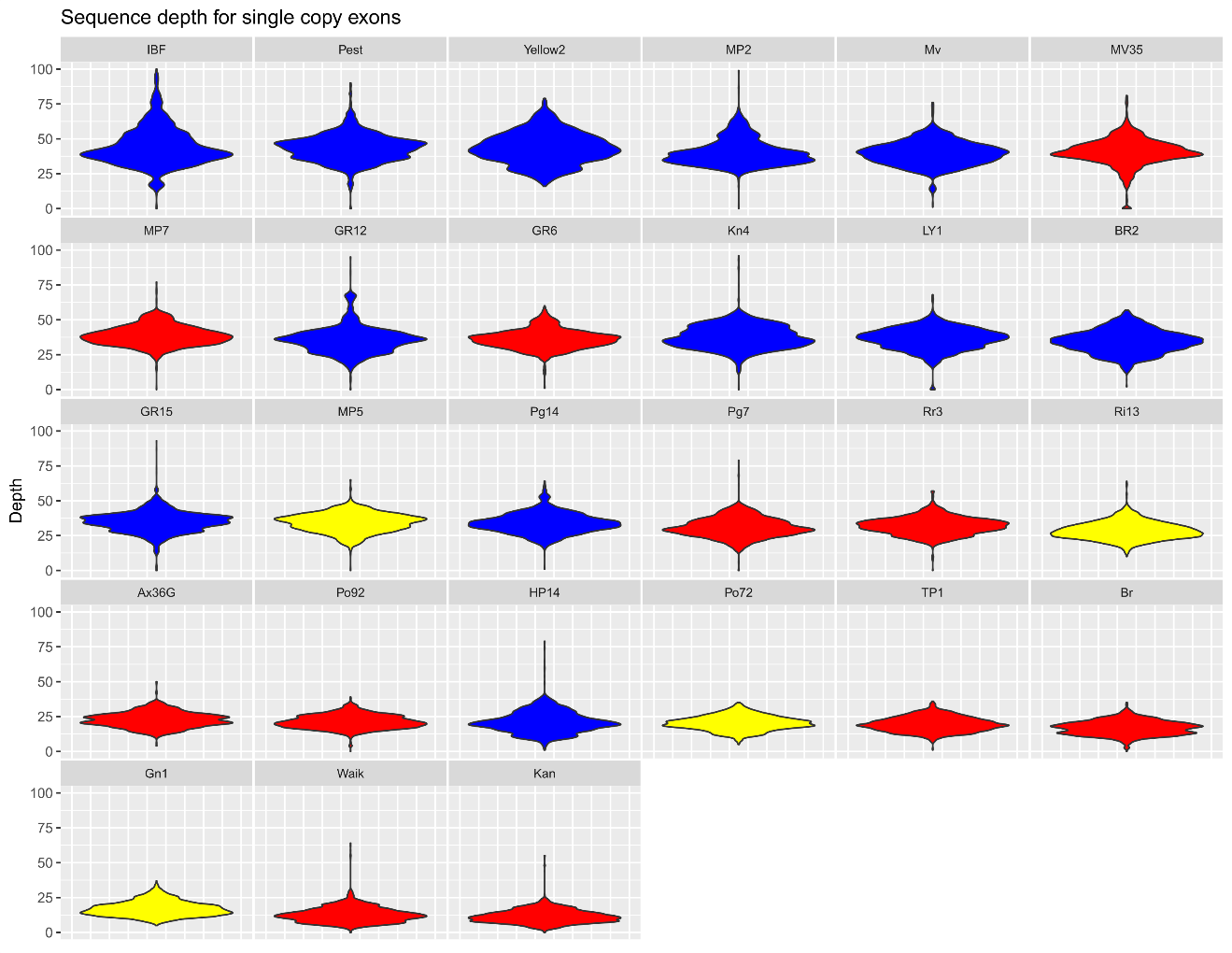


**Figure S1**: Violin plots of coverage across the 10 exons (7281 bp total) used for single-copy estimation of rDNA and histone sequences. Diploid sexual lineages represented by blue plots, triploid asexuals by red plots, and tetraploid asexuals by yellow plots. Lineages are ordered by highest to lowest median coverage values. Y-axis limited to 100 in all plots.

**
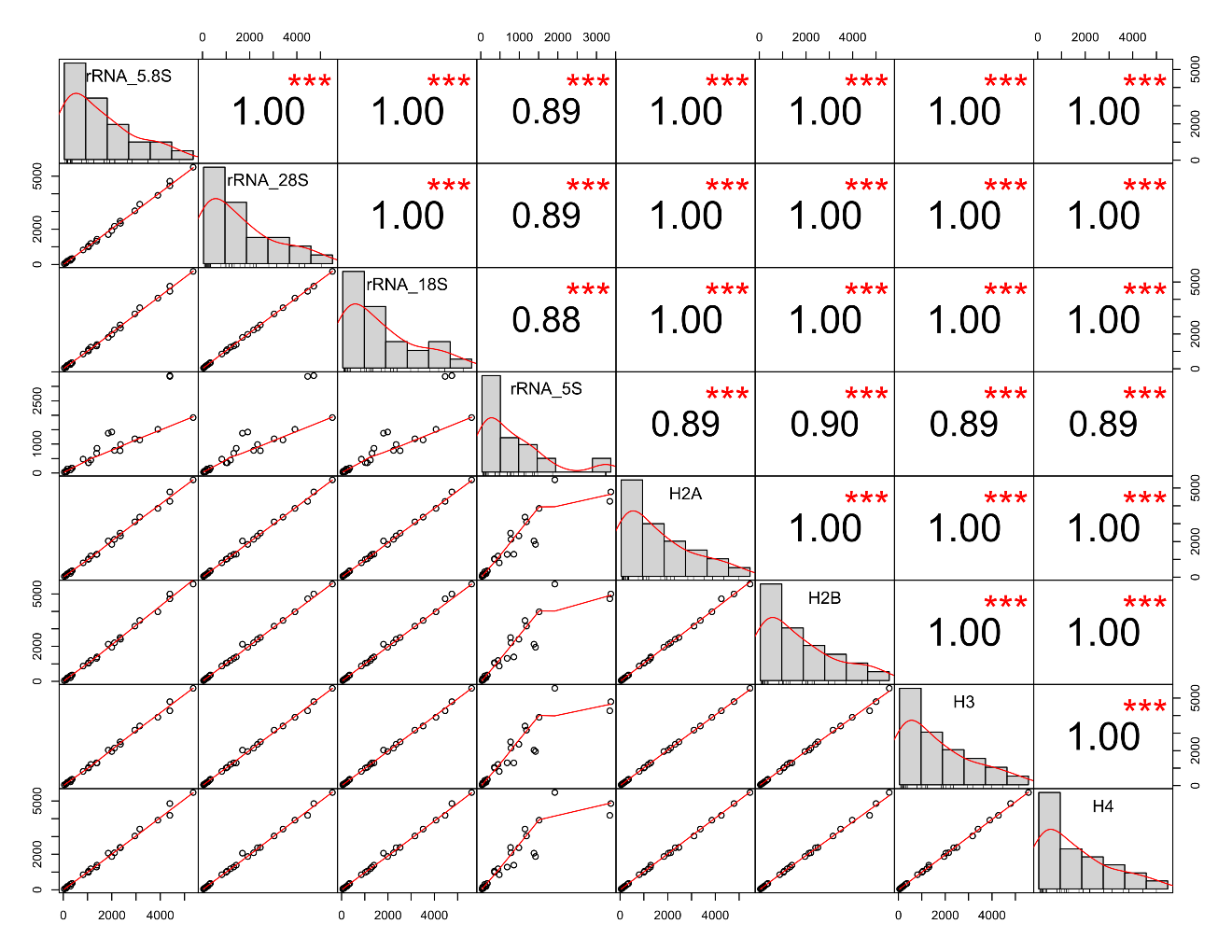
**

**Figure S2:** Copy-number estimates for each gene of the rDNA-histone locus are strongly correlated. Diagonal: distributions of copy-number values; bottom of diagonal: scatterplots with fitted lines; top of diagonal: Pearson’s r (*** = *p* < 0.001).

**
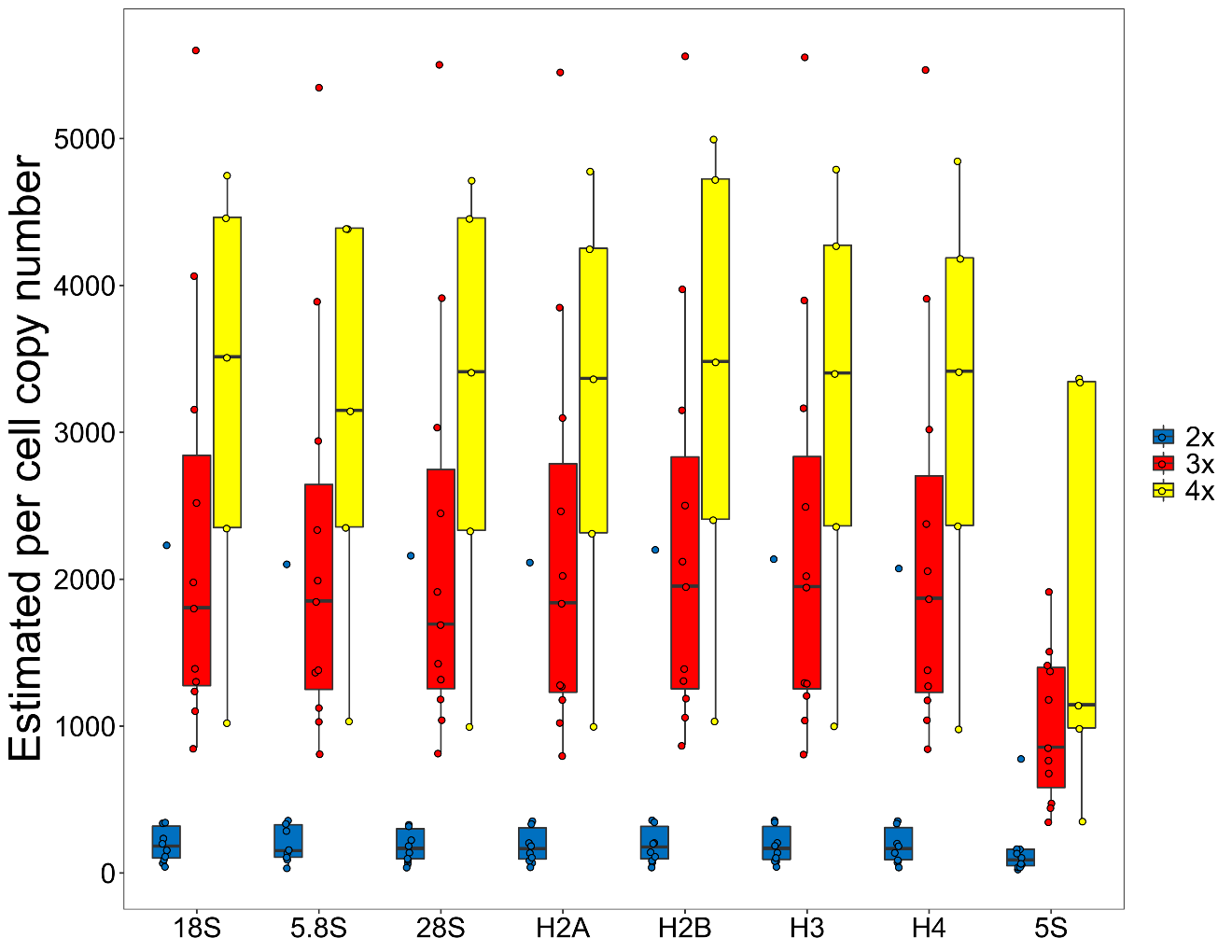
**

**Figure S3:** Asexual lineages of *P. antipodarum* have markedly higher abundance of rDNA-histone sequence in their genomes than diploid (2x) sexuals (blue) (KW adjusted *p* < 0.01). Triploid (3x, red) and tetraploid asexuals (4x, yellow) do not differ in abundance of this sequence (KW adjusted *p* > 0.5). Tukey’s boxplots with all data points shown. Estimated copy-number for each gene in the rDNA-histone array for diploid sexuals (n = 10 lineages), triploid asexuals (n = 11 lineages), and tetraploid asexuals (n = 5 lineages).

**
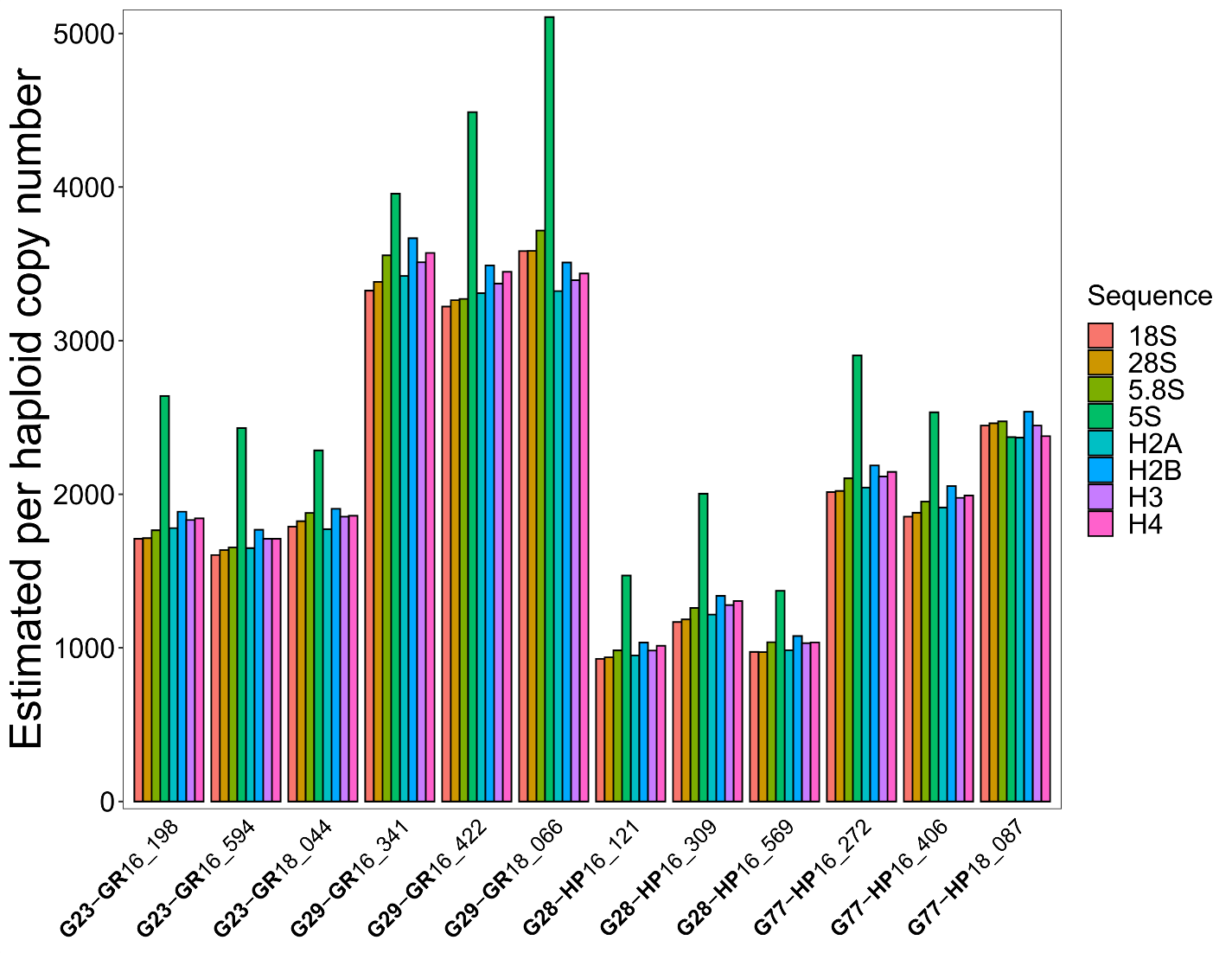
**

**Figure S4:** Estimated per-haploid copy-number for replicates of clonal lineages collected from two natural populations, Grasmere (genotypes GR23, GR29) and Haupiri (genotypes HP68, HP77). Clones (genotypes) inferred with GenoDive (Meirmans and Van Tienderen 2004) based on a 46-SNP panel. The four clones were selected from 45 clonal genotypes represented by 93 individuals from Grasmere and 19 clonal genotypes represented by 83 individuals from Haupiri. Bold text in X-axis indicates genotype (GRXX) and lake (GR = Gramsere, HP = Haupiri).

**
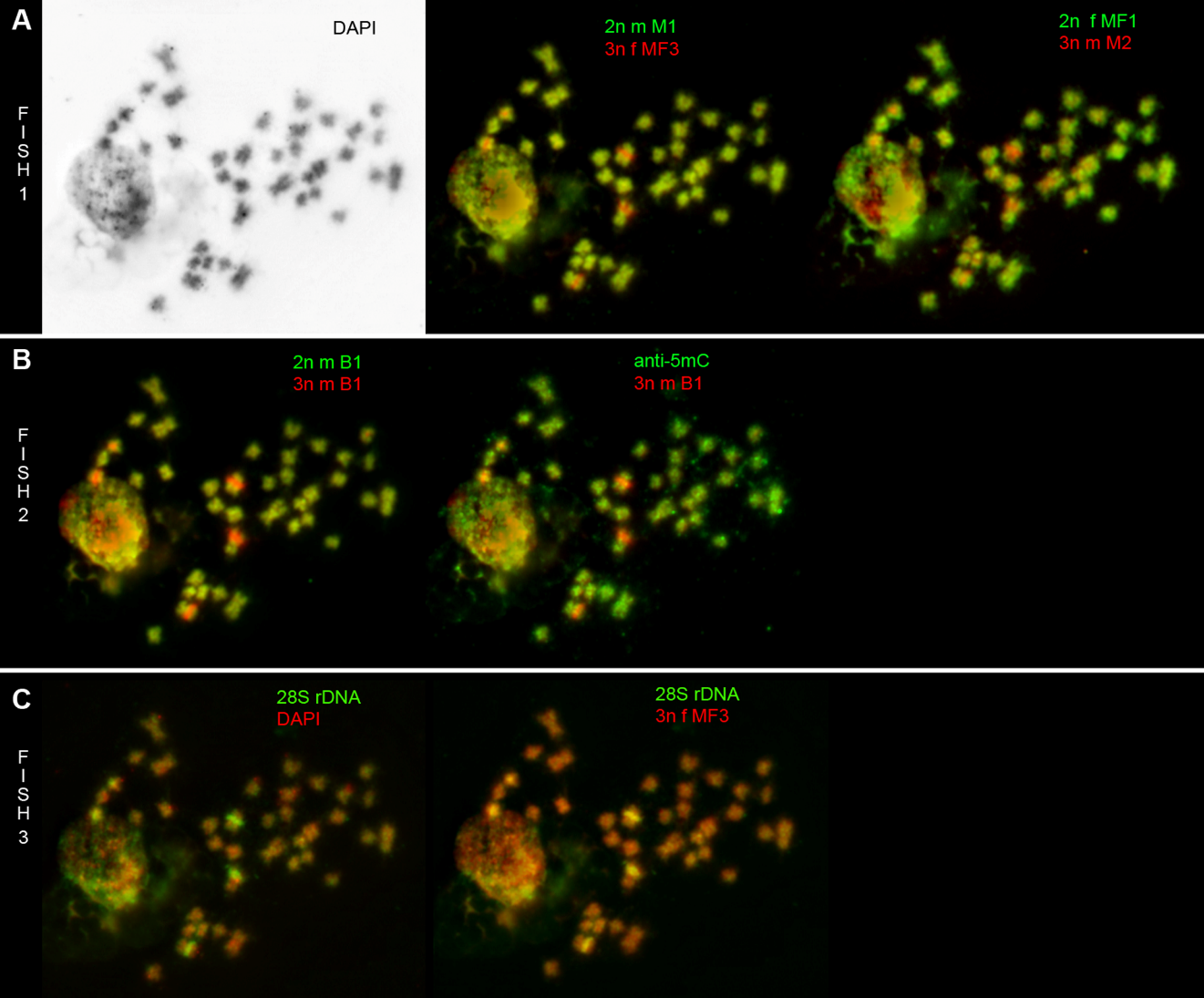
**

**Figure S5:** Example for FISH workflow employing three rounds of sequential *in situ* hybridizations to the same reference slide with triploid asexual female chromosome preparation, combined with immune-fluorescent staining of 5-methyl-cytosine residues. A) FISH round 1: CGH using a mixture of four differentially labelled genomic DNAs from *P. antipodarum,* DAPI counterstain (left), diploid sexual male M1 green vs triploid female MF3 red (center), and diploid female Mf1 green vs triploid male M2 (right), thus allowing for six different pairwise CN comparisons. B) FISH round 2: CGH using a mixture of two differentially labelled genomic DNAs, diploid sexual male B1 green vs triploid asexual male B1 red (left), combined with anti-5-mC immune-staining green, merged with gDNA from triploid asexual male B1 in red (right). C) FISH round 3: mapping of a *P. antipodarum* 28S rDNA PCR product labelled in green to DAPI-stained chromosomes shown in red (left) and co-localization analysis with regions of amplified CN by re-merging with gDNA from triploid asexual female MF3 false colored in red (right).

**
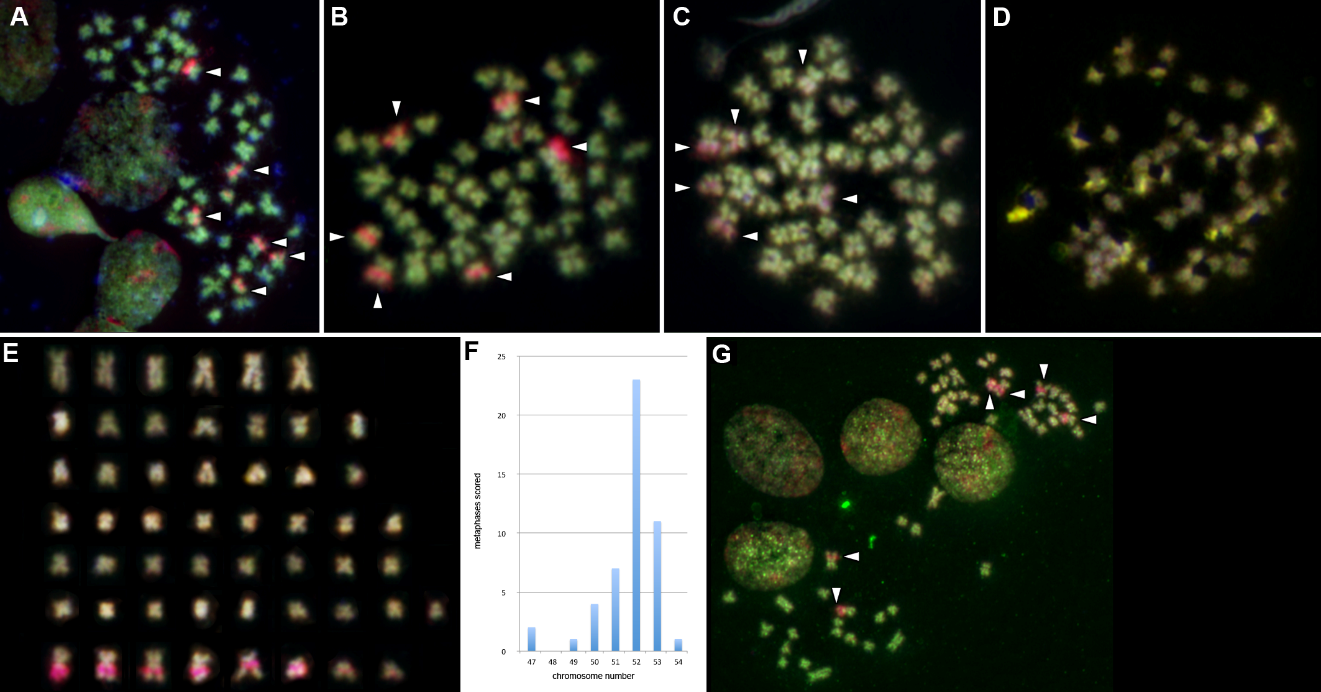
**

**Figure S6:** Additional examples for FISH experiments: A-E) FISH images from comparative genomic hybridization (CGH) experiments. A) and B) comparisons of diploid sexual individuals in green vs triploid asexual individuals in red demonstrate high CN overrepresentation in six rDNA/histone gene clusters (arrowheads) in triploids, A) 2n sexual male B1 green vs 3n asexual male B1 red, B) 2n sexual male M1 green vs 3n asexual female MF3 red. C) diploid sexual female B1 red vs diploid sexual male B1 green, each with low relatively CN as indicated by hybridization intensities similar to balanced genomic regions, though with mild CN overrepresentation in rDNA clusters in the female (red, arrowheads), D) tetraploid asexual female PA1 red vs triploid asexual female B1 green, each with relatively high CN as indicated by more intense hybridization compared to balanced genomic regions, E) karyogram of a CGH profiled cell showing 53 chromosomes, including six major and two minor rDNA/histone gene clusters (red, bottom panel). F) Chromosome counts in 49 metaphases indicate the most probable chromosome number of 3n=52 chromosomes. G) Co-localization analysis by sequential FISH and 5-mC immuno-staining mapped regions of very low DNA methylation (green) to the above-described regions of clustered CNV (red, arrowheads).
